# Glycaemic control is still central in the hierarchy of priorities in type 2 diabetes management

**DOI:** 10.1007/s00125-024-06254-w

**Published:** 2024-08-19

**Authors:** Kamlesh Khunti, Francesco Zaccardi, Aslam Amod, Vanita R. Aroda, Pablo Aschner, Stephen Colagiuri, Viswanathan Mohan, Juliana C. N. Chan

**Affiliations:** 1 Diabetes Research Centre, University of Leicester, Leicester, UK; 2 Department of Endocrinology, Nelson Mandela School of Medicine and Life Chatsmed Garden Hospital, Durban, South Africa; 3 Department of Medicine, Brigham and Women’s Hospital, Harvard Medical School, Boston, MA USA; 4 Endocrinology Unit, Javeriana University and San Ignacio University Hospital, Bogotá, Colombia; 5 Boden Collaboration, Charles Perkins Centre, University of Sydney, Sydney, NSW Australia; 6 Department of Diabetology, Dr Mohan’s Diabetes Specialities Centre and Madras Diabetes Research Foundation, Chennai, India; 7 Department of Medicine and Therapeutics, Hong Kong Institute of Diabetes and Obesity and Li Ka Shing Institute of Health Sciences, The Chinese University of Hong Kong, Prince of Wales Hospital, Hong Kong, China

**Keywords:** Cardiovascular outcomes, Glycaemic control, HbA_1c_, Legacy effect, Organ protection, Treatment paradigm, Type 2 diabetes

## Abstract

A panel of primary care and diabetes specialists conducted focused literature searches on the current role of glycaemic control in the management of type 2 diabetes and revisited the evolution of evidence supporting the importance of early and intensive blood glucose control as a central strategy to reduce the risk of adverse long-term outcomes. The optimal approach to type 2 diabetes management has evolved over time as the evidence base has expanded from data from trials that established the role of optimising glycaemic control to recent data from cardiovascular outcomes trials (CVOTs) demonstrating organ-protective effects of newer glucose-lowering drugs (GLDs). The results from these CVOTs were derived mainly from people with type 2 diabetes and prior cardiovascular and kidney disease or multiple risk factors. In more recent years, earlier diagnosis in high-risk individuals has contributed to the large proportion of people with type 2 diabetes who do not have complications. In these individuals, a legacy effect of early and optimal control of blood glucose and cardiometabolic risk factors has been proven to reduce cardiovascular and kidney disease events and all-cause mortality. As there is a lack of RCTs investigating the potential synergistic effects of intensive glucose control and organ-protective effects of newer GLDs, this article re-evaluates the evolution of the scientific evidence and highlights the importance of integrating glycaemic control as a pivotal early therapeutic goal in most people with type 2 diabetes, while targeting existing cardiovascular and kidney disease. We also emphasise the importance of implementing multifactorial management using a multidisciplinary approach to facilitate regular review, patient empowerment and the possibility of tailoring interventions to account for the heterogeneity of type 2 diabetes.

**Graphical Abstract:**

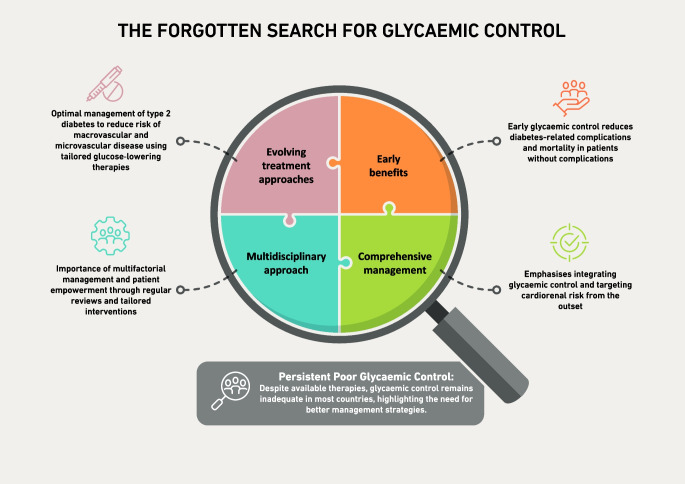

## Introduction

**Fig. 1:**
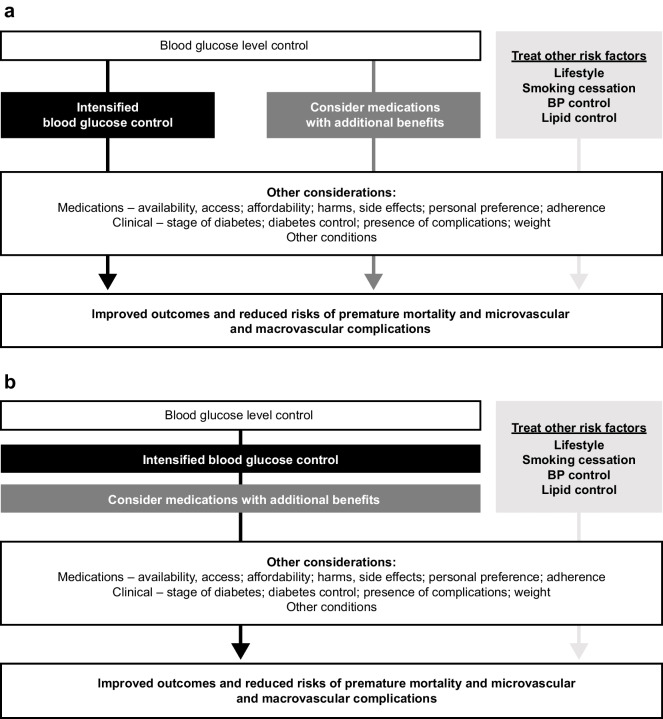
Paradigms for managing type 2 diabetes and reducing diabetes-related complications based on (**a**) current consensus recommendations by the ADA/EASD [3] and (**b**) expert opinion of the authors

During the last four decades, the number of available diabetes treatments has more than tripled. Accordingly, clinical guidelines for type 2 diabetes management have evolved from approaches focusing on glycaemic control to more holistic and individualised approaches. The 2018 ADA/EASD recommendations represented a ‘paradigm shift’ in type 2 diabetes management [1], from a primary focus on control of hyperglycaemia to a focus on therapies with specific cardiorenal protective effects without primary consideration of glucose lowering in subsets of individuals. As a result, cardiorenal protection ranks alongside glycaemic control as a key treatment target (Fig. 1a). This approach was based on the results from cardiovascular safety trials, mandated by the US Food and Drug Administration (FDA) since 2008 in response to the increased risk of myocardial infarction (MI) noted with rosiglitazone [2]. This required RCTs to demonstrate non-inferiority for cardiovascular outcomes when comparing newer glucose-lowering drugs (GLDs) with placebo on top of the best standard of care.

These post-FDA cardiovascular outcomes trials (CVOTs) enrolled individuals with (very) high cardiovascular risk profiles to facilitate the accrual of high outcome event rates (Table 1). While dipeptidyl peptidase-4 inhibitors (DPP4is) showed neutral cardiovascular effects, sodium–glucose cotransporter 2 inhibitors (SGLT2is) and glucagon-like peptide-1 receptor agonists (GLP-1 RAs) demonstrated cardiorenal benefits in individuals with existing complications or multiple risk factors. These results have led to their recommended use as second-line GLDs after metformin for organ protection in high-risk individuals [3].

**Table 1:** Key features of CVOTs and RCTs of intensive glycaemic control

Feature	CVOTs (SGLT2is and GLP-1 RAs)	Intensive glucose-lowering RCTs
Cohort generalisable to T2D	Less representative	More representative
Baseline CVD risk	High: 87%; range: 41–100%	Low: 33%; range: 3–40%
Disease duration	Longer	Shorter
Duration of trials	Shorter	Longer
Glycaemic control (active vs control)	Better	Significantly better
Microvascular outcomes assessed	Renal only	All
Macrovascular outcomes assessed	All including HF	All

HF, heart failure; T2D, type 2 diabetes

Although the long-term cardiorenal effects of these newer GLDs in individuals without cardiorenal complications and/or with few cardiometabolic risk factors have been investigated in pharmacoepidemiological studies [4], there is a lack of long-term RCTs of newer agents. Of note, in US real-world studies, around 50% of participants had clinical profiles that fulfilled none of the inclusion criteria for RCTs investigating SGLT2is, and 67% did not meet ADA recommendations for use of SGLT2is or GLP-1 RAs [5, 6]. Moreover, the absolute risk reduction for SGLT2is and GLP-1 RAs in the lower risk diabetes populations is quantitatively smaller, as the same relative hazard reduction (i.e. HR) translates into a smaller absolute risk reduction in individuals at lower risk [7, 8]. These observations raise important questions regarding the benefit of these newer organ-protective GLDs compared with the strategy of optimising glycaemic control for the majority of people with type 2 diabetes without complications.

Notwithstanding the availability of over ten classes of GLDs and the increase in the global burden of type 2 diabetes [9], there appears to be a growing perception that glycaemic control is not as important as organ-specific therapies. It is timely, therefore, to revisit the evolution of evidence supporting the importance of early and intensive blood glucose control as a fundamental strategy to optimise long-term outcomes in people with type 2 diabetes (Fig. 1b).

## Methods

Eight international diabetes experts (the authors) met virtually and in person to consider the current role of glycaemic control in the management of type 2 diabetes. Individual authors researched and provided commentaries on issues related to perceived messages and practices among physicians and barriers to evidence-to-practice translation. The authors conducted focused literature searches and identified key questions. The present article summarises key aspects of glycaemic control in people with type 2 diabetes.

## Glycaemic control and diabetes-related complications

Chronic hyperglycaemia is associated with micro- and macrovascular complications, reduced quality of life and premature mortality. The beneficial influence of glycaemic control on clinical outcomes has been shown in observational studies by the positive associations between blood glucose levels and several diabetes-related outcomes. In the UK Prospective Diabetes Study (UKPDS) 35, the incidence of complications was significantly associated with blood glucose levels, with each 10.9 mmol/mol (1%) reduction in updated mean HbA_1c_ linked to a 21% reduction in any diabetes-related endpoint, 21% reduction in diabetes-related deaths, 14% reduction in MI and 37% reduction in microvascular complications [10]. In the Swedish National Diabetes Register, an HbA_1c_ level outside the target range was the strongest predictor of acute MI and stroke [11]. Systematic reviews and meta-analysis have also indicated positive relationships between HbA_1c_ and risk of macrovascular outcomes and mortality [12].

Although the UKPDS RCT demonstrated that intensive glucose control reduced the risk of long-term micro- and macrovascular complications in people with recently diagnosed type 2 diabetes [13], three RCTs published between 2008 and 2009 showed different results in people with type 2 diabetes of long duration, the majority of whom had complications or risk factors. In the ACCORD trial, intensive glucose control increased the risk of CVD and related death [14]. In the ADVANCE trial, a more gradual approach to achieving glycaemic targets reduced the risk of the predefined combined microvascular and macrovascular outcomes [15]. In the VA Diabetes Trials, intensive glucose control did not reduce the risk of CVD, microvascular outcomes, CVD death or all-cause death [16]. Post hoc analyses generated several hypotheses around the possible reasons for this. These included a lower efficacy of intensive glucose control in older people, the presence of complications (particularly autonomic neuropathy), the risk of hypoglycaemia with intensive treatment strategies and possibly higher rates of hypoglycaemia-associated complications [16]. At the same time meta-analyses of RCTs have confirmed that intensive glucose control reduces the risk of CVD, retinopathy and nephropathy [17–20]. In this light, microvascular complications have been conclusively proven to be prevented or delayed by optimal glycaemic control in people with type 1 and type 2 diabetes [16, 19], emphasising the importance of glycaemic control as a key strategy in diabetes management.

The importance of early glycaemic control was first demonstrated in the UKPDS, in which tight control from diagnosis of type 2 diabetes reduced the MI and mortality risk 10 years later by 19–20%. In contrast, delaying glycaemic control reduced the mortality and MI risk by only 3% and 6.5%, respectively [21]. These ‘legacy effects’ became increasingly evident with prolonged follow-up. In the latest 44-year analysis of the UKPDS, early reduction of HbA_1c_ by 8.7 mmol/mol (0.8%) translated to a 10% risk reduction in diabetes-related endpoints, 17% risk reduction in MI, 26% risk reduction in microvascular complications and 10% risk reduction in mortality in the intensive treatment group compared with the control group [13]. As only a few participants in the UKPDS had CVD at baseline, a longer follow-up (i.e. more events) was required to accrue endpoints to achieve statistical significance [22]. Because the sample size and length of follow-up of a trial are dictated by the event rate (i.e. the risk of outcome occurrence), RCTs aiming for a ‘statistically significant’ result should last for several years, potentially decades, if low-risk participants have been enrolled; alternatively, longer term effects can be modelled based on the available data [23–25].

## Recognising the heterogeneity of type 2 diabetes to improve diagnosis and management

With increasing knowledge about the pathophysiology of diabetes, matching key abnormal pathways with drug mechanisms to personalise care may be the way forward compared with a ‘one size fits all’ strategy.

Age is one key aspect underpinning diabetes phenotypes. Young-onset diabetes has complex aetiologies, with both leanness and obesity being equally important, especially in non-European populations [26–28]. Epidemiological analyses have revealed an inverse association between age at diagnosis and increased risk of micro- and macrovascular complications and mortality [27], with earlier diagnosis of type 2 diabetes having a greater impact on life expectancy than late-onset type 2 diabetes [29]. The association between age at diagnosis and risk of mortality/complications is probably due to the cumulative effects of hyperglycaemia and other risk factors in addition to host-related factors, such as genetics or perinatal development [28, 30]. These data emphasise the importance of early and intensive treatment in young people with diabetes [27]. However, most RCTs (including CVOTs) have excluded younger individuals in order to attain a prespecified number of endpoints within a short period of time (i.e. greater statistical power). The lack of guidance has meant that these younger individuals are managed in a diverse manner in real-world practice, which might contribute to their poor outcomes.

The evidence is more robust in older individuals. Intensive glycaemic control is generally associated with a reduced risk of micro- and macrovascular complications, although benefits are attenuated with increasing age [11]. Treatment decisions in older people are more complex because of comorbidities, polypharmacy, frailty and cognitive dysfunction, and the benefits of intensive glycaemic control may be offset by the risk of adverse events, notably severe hypoglycaemia and falls [31, 32]. There is now consensus that glycaemic goals should be individualised with less stringent glycaemic targets in this group and that GLDs that are associated with a low risk of hypoglycaemia should be used [33]. Over-treatment to achieve stringent glycaemic goals may increase the risk of mortality in older people on insulins, sulfonylureas or glinides [34].

Alongside age, information on HbA_1c_, lipid profiles, autoantibodies, BMI, beta cell function, insulin resistance, genomics and gene expression have been variably included in models to cluster diabetes phenotypes [35]. For example, by using age, BMI, autoantibodies and markers of beta cell function and insulin resistance, individuals can be classified into five subtypes that predict insulin requirements and risk of chronic kidney disease (CKD). However, although these clusters have been replicated in European, Chinese and Indian populations, their clinical relevance has yet to be validated in other populations [36–38]. In addition, definitive evidence is needed on whether these complex clustering approaches are superior to conventional clinical and biochemical markers in informing practice [35, 39]. However, given the estimated 5% prevalence of slowly evolving autoimmune type 1 diabetes masking as type 2 diabetes, standardised measurement of autoantibodies may enable insulin-insufficient individuals to be identified, avoiding undue delays in insulin initiation [40, 41]. In support of this, variations in treatment effect across different phenotypes of type 2 diabetes have been reported in observational studies and post hoc analyses of RCTs [42]. However, pragmatic studies, ideally randomised, are needed to determine whether information on diagnostic phenotypes can be translated into therapeutic actions to improve the precision and cost-effectiveness of treatment [43].

## Impact of CVOTs on the management paradigm for type 2 diabetes

During the last few decades, a wealth of evidence has emerged from CVOTs that supports the cardiorenal protective effects of SGLT2is and GLP-1 RAs in individuals with or at (very) high risk of CVD. Such benefits are mostly independent of intensive glycaemic control and some have also been observed in people without type 2 diabetes [44–46]. These results have created a paradigm shift to a focus on organ protection with less emphasis on glycaemic control, which may have had unintended consequences for the many individuals with poor glycaemic control who do not yet have complications [3, 47]. The CVOTs included participants with CVD or multiple CVD risk factors in order to show safety first and, if demonstrated, efficacy [48] (Table 1); thus, they may not be generalisable to the wider type 2 diabetes population in routine clinical practice.

Meta-regression and subgroup analyses of CVOTs have reported similar or heterogeneous RR reductions in primary outcomes or in components of cardiorenal endpoints with SGLT2is and GLP-1 RAs when comparing individuals with vs individuals without, or with different severities of, CVD or CKD [7, 49–55]. Furthermore, given the low rate of events in individuals without complications, the same RR reduction translates to a very different absolute risk reduction, number needed to treat and cost-effectiveness [8]. These nuances highlight the need to perform baseline risk assessments to quantify the absolute benefits of SGLT2is and GLP-1 RAs. In this respect, the GRADE trial, which included participants with short-duration type 2 diabetes (<10 years) and HbA_1c_ levels of 51–69 mmol/mol (6.8–8.5%), showed that glargine and a GLP1-RA (liraglutide) were modestly better than a sulfonylurea (glimepiride) and DPP4i (sitagliptin) in reducing HbA_1c_, although all four interventions improved glucose levels [56]. More RCTs and real-world evidence are required to assess the absolute benefits of the different GLD classes in low-risk individuals with short disease duration (see Text box: ‘Current evidence gaps and avenues for future research’).


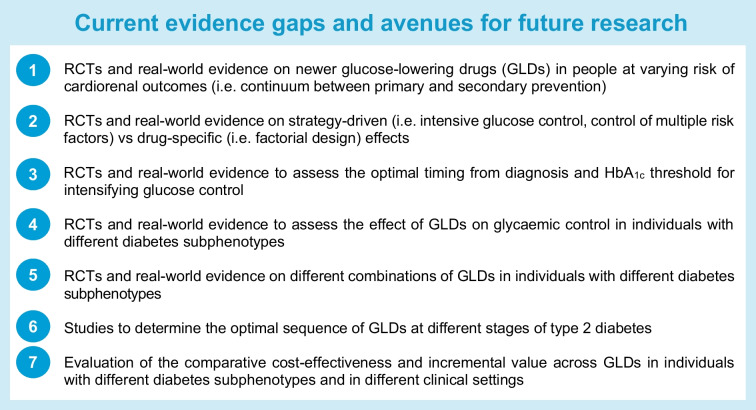


In all placebo-controlled CVOTs, participants in the active treatment groups achieved lower HbA_1c_ than those in the control groups, suggesting that glycaemic control might have contributed to the positive outcomes [51, 57, 58] (Table 1). A post hoc analysis of the LEADER trial (which compared the risk of major adverse cardiovascular events [MACE] between liraglutide and placebo) suggested that up to 41% and 83% of the cardiovascular benefits of liraglutide (depending on the statistical method used) were mediated by a reduction in HbA_1c_ [57]. On the other hand, a mediation analysis of the EMPA-REG OUTCOME trial (which compared the risk of MACE between empagliflozin and placebo) identified haematocrit markers as potential mediators, with a smaller mediating role of glycaemic control, in line with the mechanisms of action of SGLT2is in regulating sodium and water metabolism [59]. A meta-regression of CVOTs estimated that if a 9.8 mmol/mol (0.9% ) reduction in HbA_1c_ was achieved instead of the observed mean of ~0.4% (~4.4 mmol/mol), the reduction in MACE would have been approximately 33%. These findings argue that glycaemic control may contribute, to a variable extent, to the effects of SGTL2is and GLP-1 RAs on MACE [51]. Furthermore, real-world data suggest that different cardiometabolic risk factors might be related to different clinical outcomes. For example, lipid and BP levels might have a greater effect size on MI/ischaemic heart disease, while body weight and glycaemic control might be more relevant for stroke, heart failure and kidney disease [60, 61]. In the CVOTs of DPP-4is, GLP-1RAs and SGLT2is, the risk reduction in non-fatal stroke was entirely driven by glycaemic control [62]. Taken together, there is a need to gather more evidence to study the cost-effectiveness across all available GLDs of improving individual outcomes, especially in those without complications, guided by their baseline risk factors (see Text box: ‘Current evidence gaps and avenues for future research’).

In a typical primary care setting, only one-third of people with type 2 diabetes have CVD [63]. For those without CVD, physicians have a clear window of opportunity to focus on achieving and maintaining optimal glycaemic control alongside the control of CVD risk factors to prevent organ damage. Moreover, hyperglycaemia is a causal factor for microvascular complications, which is associated with poor quality of life and increased risk of CVD, for which optimal glycaemic control remains a definitive solution [64, 65].

## Glycaemic control as part of holistic multifactorial risk factor management

The overall goals of care in all people with type 2 diabetes centre on minimising the disease burden by reducing complications and premature mortality while maximising quality of life. This is achieved through the provision of personalised, evidence-based, cost-effective, accessible and affordable holistic interventions to improve blood glucose levels and risk factor control. Therapeutic strategies should aim for intensive glucose lowering to achieve personalised glycaemic targets, especially in people with early type 2 diabetes who do not have complications, rather than being based exclusively on organ-protective effects.

Traditional drugs such as metformin, sulfonylureas and DPP4is have proven glucose-lowering efficacy in RCTs and are well tolerated in real-world settings. Combination therapy can be used to achieve early and sustained optimal glycaemic control instead of the more common stepwise introduction of additional therapies. The VERIFY RCT demonstrated that early combination of metformin and a DPP4i delayed treatment escalation compared with incremental use of medications [66]. Similarly, population-based real-world evidence showed that early treatment escalation with DDP4is (within 2 years of diagnosis) on a background of metformin and sulfonylurea was associated with a delay in insulin initiation and a reduction in cardiorenal events and all-cause death [67, 68].

It is important to note that, in the CVOTs of newer GLDs, including SGLT2is and GLP-1RAs, most participants were treated with conventional GLDs as well as renin–angiotensin–aldosterone system inhibitors, statins and antiplatelet therapy. The clustering of type 2 diabetes with other cardiometabolic risk factors emphasises the need for multifactorial risk factor management. In the Steno-2 study, intensive risk factor management (blood glucose, BP, lipids) with medications and lifestyle changes (smoking cessation) not only reduced the risk of microvascular complications but also translated into long-term reductions in cardiorenal events and mortality risk [69–71]. Several other studies have provided convincing evidence that intensive control of multiple risk factors reduces cardiorenal endpoints and mortality risk at all stages of diabetes [72–74].

People with type 2 diabetes have diverse needs beyond medical multifactorial risk management. The latest ADA/EASD guidelines [3] highlight the importance of identifying social determinants of health, including socioeconomic status, physical environment, food insecurity/access, healthcare access, affordability and quality, and social context [75]. While some of these factors might not be modifiable, clinicians are in a position to advocate and engage relevant stakeholders to provide holistic care to address the physical, mental, behavioural and social needs of their patients and improve their outcomes. As holistic, patient-centred and value-based care is context-dependent, using a multidisciplinary team approach is an effective strategy to address the multiple needs of people with diabetes. The allied healthcare professionals/workers making up such teams provide the much-needed liaison between patients and doctors to improve communication and relationships [76]. With the increasing use of electronic medical records, the systematic and ongoing collection of data by establishing well-designed registers that document upstream and modifiable risk factors, with regular linkage to medications, laboratory results, hospitalisations and deaths, can be extremely valuable. This can help inform decisions at both personal (patients and practitioners) and policy levels to improve system- and personal-level healthcare delivery aimed at reducing the burden of diabetes and its complications [77].

## Unanswered questions regarding the generalised use of new GLDs

With the growing burden of type 2 diabetes in emerging countries, the choice of GLDs should also be considered in the context of available resources. Although generic SGLT2is are beginning to be available, newer SGLT2is and GLP-1 RAs continue to emerge that are significantly more expensive than traditional GLDs. In these circumstances, GLDs with long-established effectiveness, safety and affordability remain important therapeutic options [78].

In individuals with established complications, organ-protective SGLT2is and GLP-1 RAs reduce the risk of cardiorenal outcomes [79]; however, their effects on microvascular complications, notably neuropathy and retinopathy, have not been extensively explored [44]. Moreover, their efficacy against individual components of MACE is not uniform: while GLP-1 RAs reduce the risk of stroke [80, 81], SGLT2is mainly reduce the risk of hospitalisations for heart failure and the risk of adverse kidney outcomes [51, 52, 79, 81–83]. Furthermore, the effect size of GLP-1 RAs and SGLT2is for various outcomes differs within the same class of drugs and varies according to individual risk profiles [84, 85]. Although these observations suggest the potential complementary beneficial effects of these two drug classes, the cost-effectiveness of this combination compared with early attainment of multiple treatment goals remains to be confirmed [25, 86, 87].

All GLDs are effective in reducing HbA_1c_, with baseline HbA_1c_ being the main determinant of the reduction [88]. However, for all GLDs there is considerable variation in the magnitude of effect between and within drugs classes [7, 81, 89–94]. Similarly, the different mechanisms of actions of GLDs result in different safety profiles, both within and between drug classes. These include increased risk of hypoglycaemia (potentially life-threatening) for sulfonylureas and insulin [95, 96], gastrointestinal side effects for GLP1-RAs [89, 97], urogenital sepsis for SGLT2is [90, 97], and heart failure and altered bone metabolism for thiazolidinediones [98]. Further head-to-head RCTs are required to better elucidate the efficacy/safety profiles of these medications and guide decisions based on the overall drug profiles and individuals’ concurrent metabolic abnormalities to maximise efficacy and minimise harm, while taking into consideration individual preference and affordability.

## Barriers to improving type 2 diabetes management

Optimal glycaemic control remains a universal care gap. A systematic review of observational studies published between 2020 and 2022 reported that 45–93% of people with type 2 diabetes had poor glycaemic control, with considerable inter- and within-country variations [99]. These variations may be due to many factors, such as late diagnosis, delayed intervention, suboptimal self-management and limited access to care and effective treatments. In this light, glycaemic control has not improved over time despite the introduction of many GLDs and diabetes technologies [100].

Compared with RCTs, the magnitude of the HbA_1c_ reduction achieved with GLDs is lower in real-world clinical practice [101]. Among the contributing factors, poor medication adherence accounts for approximately 70% of the discrepancy between RCTs and real-world settings (Table 2) [27]. Therapeutic inertia (i.e. the delay in initiating/modifying treatment) is a key barrier to optimal type 2 diabetes management; delaying treatment intensification means missed opportunity to reduce adverse clinical outcomes [102]. However, some strategies implemented at provider (i.e. measurement of inertia through audits), patient (i.e. reminders through text messaging) and system (i.e. structured education sessions) levels have resulted in a reduction in therapeutic inertia [103].

**Table 2:** Determinants of treatment adherence in people with type 2 diabetes and potential causes of therapeutic inertia

Determinants of treatment adherence in people with type 2 diabetes	Potential causes of therapeutic inertia
Patient-related: • Sociodemographic - Age and gender - Education/health literacy - Income/socioeconomic status - Home location (rural or urban) • Risk factors - Disease duration - Other risk factors (e.g. BP, lipids, obesity) or comorbidities • Health behaviour and self-management - Use of tobacco and alcohol - Diet and exercise - Sleep and stress - Self-monitoring (e.g. blood glucose, BP, body weight) • Cognitive-psychosocial factors - Diabetes knowledge - Poor memory - Depressive symptoms - Beliefs about illness - Satisfaction with treatment - Relationship with healthcare team - Family support	Provider-related: • Inexperience with managing the condition and/or treatment intensification, such as initiation of insulin or other injectables followed by titration and maintenance • Concern about side effects, such as hypoglycaemia or weight gain • Lack of time or resources to provide patient education and empowerment to promote self-management and the treatment adherence needed to attain treatment goals early • Insufficient communication and engagement with patients regarding the short- and long-term benefits of treatment and how to reduce side effects of treatment
Treatment-related: • Availability of medications • Number of medications • Types of medication (e.g. side effects, regimen complexity, requirement for injection) • Cost of medications	Patient-related: • Poor health literacy with insufficient understanding of the nature of diabetes, its complications and the need for treatment intensification • Concern about side effects, such as hypoglycaemia or weight gain • Fear of needles or reluctance to self-inject or to self-monitor blood glucose • Psychological resistance, anxiety, depression, frustration or discouragement • Limited ability to pay for medications • Lack of ongoing support
Healthcare system-related: • Regularity of follow-up • Regularity of assessment of risk factor control and complications • Adequacy of education and support from healthcare team • Access to community-based resources (e.g. diabetes educators, lay associations) • Continuation of care • Adherence to guidelines	Healthcare system-related: • Availability and cost of new medications • Insufficient insurance coverage and need for co-payments • Lack of specialist nurses, diabetes educators or psychological support • Lack of integration among healthcare providers with overlaps and omissions in care processes • Lack of protocol or workforce to implement regular assessments to detect silent risk factors and complications (e.g. eye, feet, blood, urine) for early intervention and to close care gaps • Lack of relevant local/regional guidelines or clinical trial/real-world evidence for specific populations (e.g. young, non-European or low-risk individuals)

In low-/middle-income countries, the availability of, and access to, medications are also major barriers. In all countries, variations in the costs of drug acquisition and administration, differences in reimbursement schemes and irregularity in pricing means that even cheap generic medications can become unaffordable. The WHO includes metformin, sulfonylureas, insulin and SGLT2is in the list of essential medications. To this end, there is an urgent need for policymakers to align the interests of all stakeholders and implement context-relevant drug financing policies to ensure that individuals have access to these life-saving medications.

## Conclusions

Despite the central importance of early glycaemic control to improving outcomes throughout the lifespan of people with diabetes, real-world data show that glycaemic control remains poor in most settings. Most people with diabetes will benefit from early achievement and maintenance of glycaemic control. In these individuals, all GLD classes have been demonstrated to be safe and effective in achieving glycaemic targets, especially if supported by a self-management programme with regular assessment and control of risk factors. In addition to improving glycaemic control, in individuals with or at (very) high risk of cardiorenal complications, early use of GLDs with organ-protective effects should be considered if accessible and affordable, although many people will still need additional GLDs and other therapies to achieve multiple treatment targets. Each person with diabetes has a unique profile, which calls for individualised and holistic management beyond medications. By reorganising settings, workforces and models of care, it is possible to exercise a team approach to gather data regularly and stratify risk, empower self-care, reduce therapeutic inertia and use available multiple tools effectively to improve long-term outcomes.

## Data Availability

Data sharing is not applicable to this article as no datasets were generated or analysed during the current study.

## Abbreviations

CKD: Chronic kidney disease
CVOT: Cardiovascular outcomes trial
DPP4i: Dipeptidyl peptidase-4 inhibitor
FDA: Food and Drug Administration
GLD: Glucose-lowering drug
GLP-1 RA: Glucagon-like-peptide-1 receptor agonist
MACE: Major adverse cardiovascular events
MI: Myocardial infarction
SGLT2i: Sodium–glucose cotransporter 2 inhibitor
UKPDS: UK Prospective Diabetes Study

## Authors’ relationships and activities

KK is supported by the National Institute for Health and Care Research (NIHR) Applied Research Collaboration East Midlands (ARC EM) and the NIHR Leicester Biomedical Research Centre (BRC) and has served as a consultant or speaker for, or received grants for investigator-initiated studies from AstraZeneca, Bayer, Novartis, Novo Nordisk, Sanofi-Aventis, Eli Lilly, MSD, Boehringer Ingelheim, Oramed Pharmaceuticals, Roche and Applied Therapeutics. FZ has received consulting fees from Daiichi Sankyo. VM has received research or educational grants from Abbott, Bayer, Boehringer Ingelheim, Eli Lilly, LifeScan, MSD, Novartis, Novo Nordisk, Sanofi-Aventis, Servier, USV, Dr. Reddy’s, Sun Pharma, Intas, Lupin, Glenmark, Zydus, Ipca, Torrent, Cipla, Biocon, Primus, Franco-Indian, Wockhardt, Emcure, Mankind, Medtronic, Fourrts, Apex, GSK and Alembic. PA has received honoraria or consulting fees from Boehringer Ingelheim, Janssen, MSD, Novartis and Sanofi and has participated in company-sponsored speakers bureaus for Eli Lilly, Boehringer Ingelheim, MSD, Novartis, Novo Nordisk and Sanofi. JCNC has received grants through her institutions and personal fees for consultancy and lectures from Astra Zeneca, Bayer, Boehringer Ingelheim, Hua Medicine, Eli Lilly, Merck, MSD, Powder Pharmaceuticals, Sanofi, Viatris and Zuellig Pharma outside the submitted work. SC has received an honorarium from Servier. The authors declare that there are no other relationships or activities that might bias, or be perceived to bias, their work.
